# Efficacy and volume stability of a customized allogeneic bone block for the reconstruction of advanced alveolar ridge deficiencies at the anterior maxillary region: a retrospective radiographic evaluation

**DOI:** 10.1007/s00784-023-05015-0

**Published:** 2023-04-14

**Authors:** Oliver Blume, Michael Back, Elek Dinya, Daniel Palkovics, Peter Windisch

**Affiliations:** 1 Private Practice Dres. Back & Blume, Tal 13, 80331 Munich, Germany; 2 Institute of Digital Health Sciences, Faculty of Public Services, Semmelweis University, Ferenc sqr. 15, Budapest, 1094 Hungary; 3 Department of Periodontology, Semmelweis University, Szentkirályi str. 47, Budapest, 1088 Hungary

**Keywords:** Customized allogeneic bone block, Alveolar ridge augmentation, 3D evaluation, Volume stability

## Abstract

**Objectives:**

The aim of this retrospective case series was to evaluate the efficacy and volume stability of a customized allogeneic bone block (CABB) for the hard tissue reconstruction of severely atrophied anterior maxillary ridges.

**Materials and methods:**

Hard tissue alterations between baseline (T1), 2-month follow-up (T2), and 6-month follow-up (T3) cone-beam computed tomography scans were evaluated with semi-automatic segmentation. Following automatic spatial alignment of the datasets, 3D subtraction analysis was performed. The volume stability of the inserted allogeneic bone block was determined on the basis of the ratio of the T3 and T2 hard tissue volumes.

**Results:**

The newly formed hard tissue volume at T2 averaged at of 0.75 cm^3^ ± 0.57 cm^3^, whereas at T3, an average of 0.52 cm^3^ ± 0.42 cm^3^ volumetric hard tissue gain could be detected. The T3/T2 ratio was found to be 67.83% ± 18.72% on average. The dice similarity coefficient between the T2 and T3 hard tissue models averaged at 0.73 ± 0.15.

**Conclusions:**

Cancellous CABBs are a reliable option for the reconstruction of severely atrophied alveolar ridges. The resorption rates of these grafts are similar to those found in the literature; however, with precise manufacturing and proper intraoperative flap management, the resorption rates may be reduced.

**Clinical relevance:**

With precise knowledge of the resorption patterns, the shape of blocks can be altered in the future to compensate for the volumetric loss.

**Supplementary Information:**

The online version contains supplementary material available at 10.1007/s00784-023-05015-0.

## Introduction

The development of surgical and prosthetic solutions in implant dentistry has led to a state where there are multiple scientifically proven treatment options for almost any clinical scenario [1]. Implant-born prosthetic solutions are even feasible at edentulous areas presenting severe alveolar hard tissue impairment. Different surgical approaches can be utilized to reestablish sufficient hard tissue mass necessary for implant placement. Surgical techniques include distraction osteogenesis, ridge splitting, guided bone regeneration (GBR), and onlay grafting [2, 3]. Onlay grafting utilizing intra- or extraoral block grafts and staged GBR yield similar results both in terms of the complication rate and implant survival rate [4]. In cases of severely atrophied alveolar ridges, intraoral autogenous bone blocks may not present sufficient volumes; therefore, large bone grafts must be harvested from extraoral donor sites (e.g., iliac crest or calvaria). The necessity of hospitalization, unpredictable premature graft resorption, and donor site morbidity are the most common complications of OG utilizing extraoral donor sites [5]. Various studies have reported different graft resorption rates [6–17]. Contradictory data are attributed to differences in the observation period, type of reconstruction, use of provisional dentures, and location of the donor site among studies. Despite literature inconsistencies, different authors agree that (i) the greatest bone resorption occurs in the first year after alveolar ridge augmentation; (ii) there are significant differences between bone blocks harvested from different donor sites (calvarial grafts yield lower resorption rates than do iliac grafts); (iii) grafts should be oversized to compensate for the resorption; (iv) the use of corticocancellous blocks is advised; and (v) removable dentures should be avoided at the surgical area, as they may cause complete resorption of the graft [16].

As an alternative to autogenous block grafts, allogeneic grafts can be used for the reconstruction of highly atrophied alveolar ridges. Thereby, the preparation of a second surgical site and the occurrence of a donor site complication can be completely avoided, reducing the invasiveness of the procedure. In the systematic review by Monje and co-workers [18] summarizing data of 15 articles, the mean resorption rate of allogenous bone blocks was found to be lower (mean, 21.7%) than that of autogenous bone blocks. Allogeneic bone blocks are hence favored not only by patients owing to reduced invasiveness and the non-necessity of hospitalization but also by clinicians owing to the reduced amount of unwanted graft resorption. Wang and co-workers [19] recently compared the efficacy between customized allogeneic bone blocks (CABBs) and autogenous bone blocks for alveolar ridge augmentation. They reported less bone resorption with customized allogeneic bone grafts than with autogenous bone grafts and emphasized the importance of three-dimensional (3D) technology for an elaborative preoperative planning. Based on preoperative cone-beam computed tomography (CBCT) scans, CABBs are created using computer-aided design/computer-aided manufacturing (CAD/CAM) technologies. With CAD/CAM, high-accuracy CABBs can be manufactured; therefore, intraoperative manual adjustments are largely unnecessary, reducing the duration of the procedure, risk of graft contamination, and pressure applied by soft tissues owing to the more anatomical shape [20, 21]. In addition, the space between the grafts and recipient alveolar ridges is minimal, resulting in a larger contact surface, which is an ideal support for graft revascularization and rapid integration [22–24]. This is of particular importance for complex bone defect morphologies [24].

To date, the literature on the resorption patterns and 3D morphological alterations of CABBs following hard tissue reconstruction is limited. Hence, the aim of this retrospective case series was to evaluate the efficacy and volume stability of CABBs for the hard tissue reconstruction of severely atrophied anterior maxillary ridges.

## Materials and methods

### Null hypothesis

Our hypothesis was that in terms of volumetric heart tissue gain and resorption rate, similar results can be achieved with the application of CABBs achieved as with autogenous bone blocks.

### Study design

In this retrospective study, 23 patients presenting severe combined horizontal and vertical alveolar ridge defects in the anterior maxillary region treated with a cancellous CABB (maxgraft bonebuilder, botiss GmbH, Zossen, Germany) were evaluated. The participants were treated at the Back & Blume private dental office with a customized CAD/CAM allogeneic bone block between 2017 and 2020. The study was conducted in full accordance with the revised Declaration of Helsinki (2013) [25] and was approved by our local ethics committee (Ethical Committee of Ludwig-Maximilians-University Munich, Germany; approval number: 18-898). A signed written informed consent was acquired from all participants.

### Patient selection

The inclusion criteria were as follows: (i) absence of general medical conditions ( previous irradiation therapy in the head and neck region within the last 2 years, uncontrolled diabetes, systemic steroid treatment, or systemic bisphosphonate treatment); (ii) non-smoking status; (iii) full mouth plaque score of ≤25% [26]; (iv) full mouth bleeding score of ≤25% [27]; and (v) healed alveolar ridge defects unfitted for implant placement with or without simultaneous hard tissue augmentation.

**Fig. 1:**
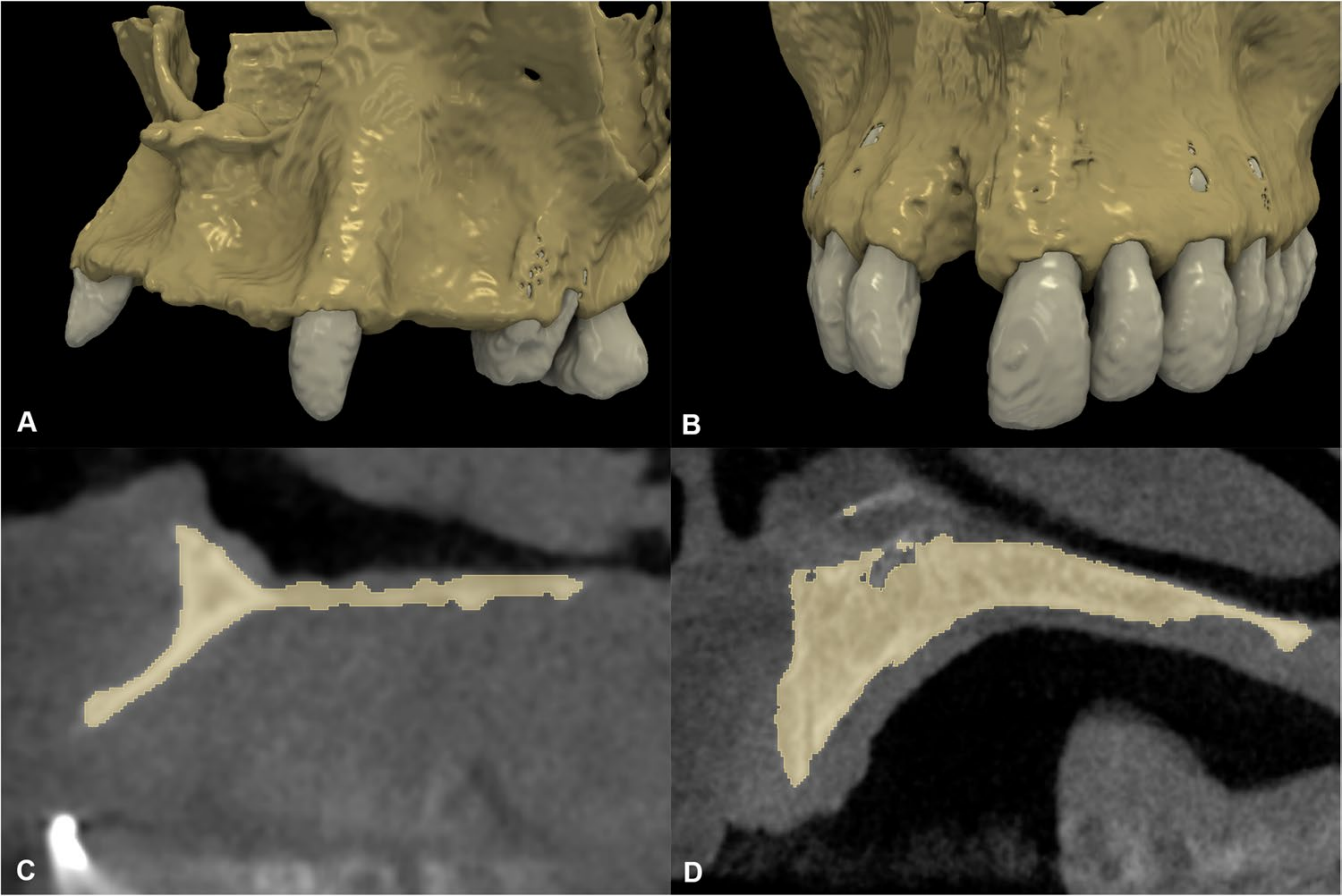
Baseline radiographic images. **A** 3D reconstruction with semi-automatic segmentation of an alveolar ridge defect at a multiple-tooth gap. **B** 3D reconstruction with semi-automatic segmentation of an alveolar ridge defect at a single-tooth gap. **C** Sagittal view of the baseline ridge dimensions at a multiple-tooth gap. **D** Sagittal view of the baseline ridge dimensions at a single-tooth gap

**Fig. 2:**
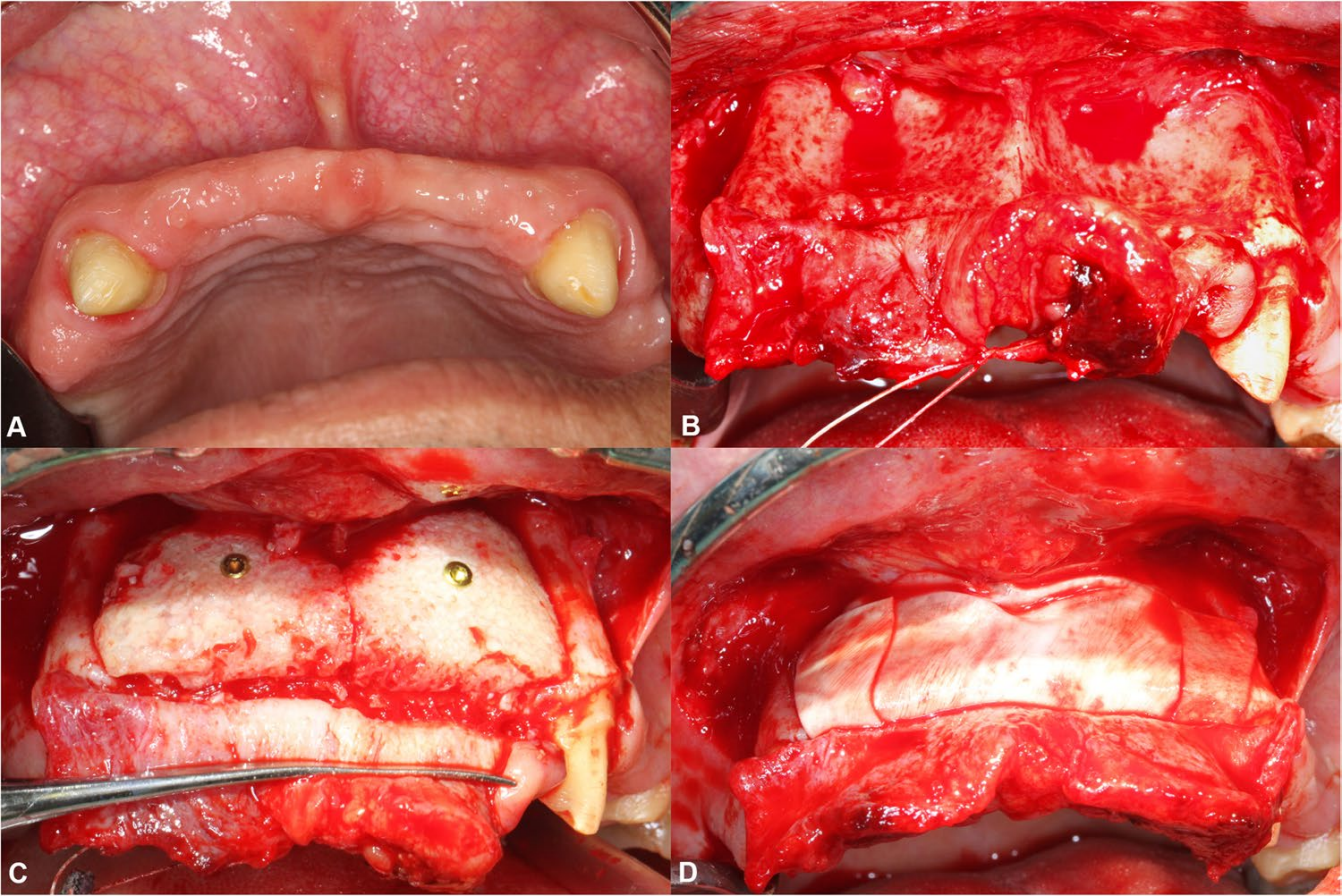
Alveolar ridge augmentation procedure. **A** Baseline clinical situation. **B** Intraoperative alveolar ridge morphology at baseline. **C** Customized allogeneic bone block fixed to the alveolar ridge with osteosynthesis screws. **D** Bone blocks covered with a long-term resorbable pericardium membrane

The exclusion criteria were as follows: (i) prolonged antibiotic/anti-inflammatory therapy prior to surgery, (ii) substance abuse, (iii) pregnancy or lactation, (iv) under 18 years of age, and (v) post-extraction defects and tooth extraction within 3 months prior to hard tissue augmentation (Figs. 1 and 2A).

### Data acquisition and surgical planning

CBCT scans were obtained at three different time points: baseline (T1), 2-month follow-up (T2), and 6-month follow-up (T3). They were taken at the following parameters using an I-CAT FLX CBCT machine (KaVo Dental GmbH, Bieberach an der Riß, Germany): (i) voxel size (0.250 μm), (ii) X-ray tube current (4–7 mA), (iii) voltage (120 kVp), and (iv) field of view (16.5 × 13.5 cm) [28]. Digital Imaging and Communications in Medicine images of the baseline CBCT datasets were imported into a digital planning software (coDiagnostiX, version 10.2.0.15659, Dental Wings Inc., Montreal, Canada) for the assessment of the baseline alveolar ridge defect morphology and planning of the CABB. The baseline alveolar ridge defects were classified at each implantation site according to the HVC ridge deficiency classification [29]. Following planning, a CABB was manufactured from the femoral heads of living donors who underwent arthroplastic surgery (maxgraft bonebuilder, botiss biomaterials GmbH, Berlin, Germany). For the prevention of pressure and eventual graft resorption caused by the protruding screw head, a countersink was incorporated into the design of the blocks.

### Surgical treatment protocol

#### Flap elevation

The bone augmentation procedures were conducted under general anesthesia. The extent of the edentulous area did not affect the surgical modality. Flap preparation on the buccal aspect was conducted on the basis of the semi-pillar incision design introduced by our group [22]. A horizontal incision was created on the buccal aspect within the mobile mucosa approximately 20 mm apically from the midcrestal line. Thereafter, a single vertical releasing incision was created at the distal aspect of the surgical area. A unilateral full-thickness mucoperiosteal flap was subsequently elevated on the buccal aspect, while the keratinized mucosa on the crestal and palatal aspects remained attached to the underlying bone (Fig. 2B).

#### Cancellous allogeneic bone block fixation

Prior to block positioning, the cortical layer at the augmented site was perforated using a diamond bur to induce bleeding for an enhanced vascularization of the graft. Following the hydration of the graft, the allogeneic bone block was placed onto the recipient site without any further adjustments. Block fixation was conducted using titanium osteosynthesis screws (Medartis AG, Basel, Switzerland) (Fig. 2C). Thereafter, the area was covered with a long-term resorbable porcine pericardium membrane (Jason membrane, botiss biomaterials GmbH, Zossen, Germany) and was fixated with titanium pins (Fig. 2D) for additional barrier function. Tension-free wound closure was achieved with single interrupted sutures utilizing 4-0 and 5-0 resorbable suturing materials (Vicryl Rapide, Ethicon, Raritan, New Jersey, USA). The sutures were removed after 14 days.

#### Re-entry procedure and implant placement

**Fig. 3:**
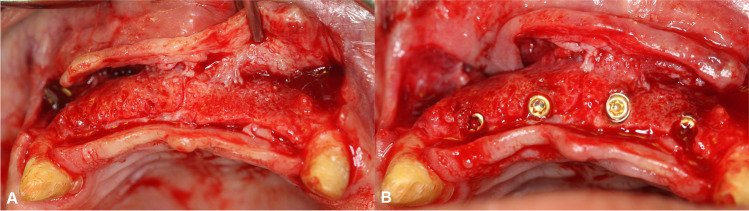
Re-entry surgery and implant placement. **A** Intraoperative alveolar ridge morphology at implant placement. **B** Implants placed at the reconstructed alveolar ridge

Following a 6-month healing period, guided implant placement was planned on the T3 CBCT scan. Direct evaluation of the reconstructed alveolar ridge, removal of the block fixation screw, and placement of a dental implant were performed during a re-entry procedure. Further hard tissue augmentation was not necessary in any of the cases (Fig. 3).

### Radiographic evaluation of hard tissue changes

#### Segmentation—3D model acquisition

**Fig. 4:**
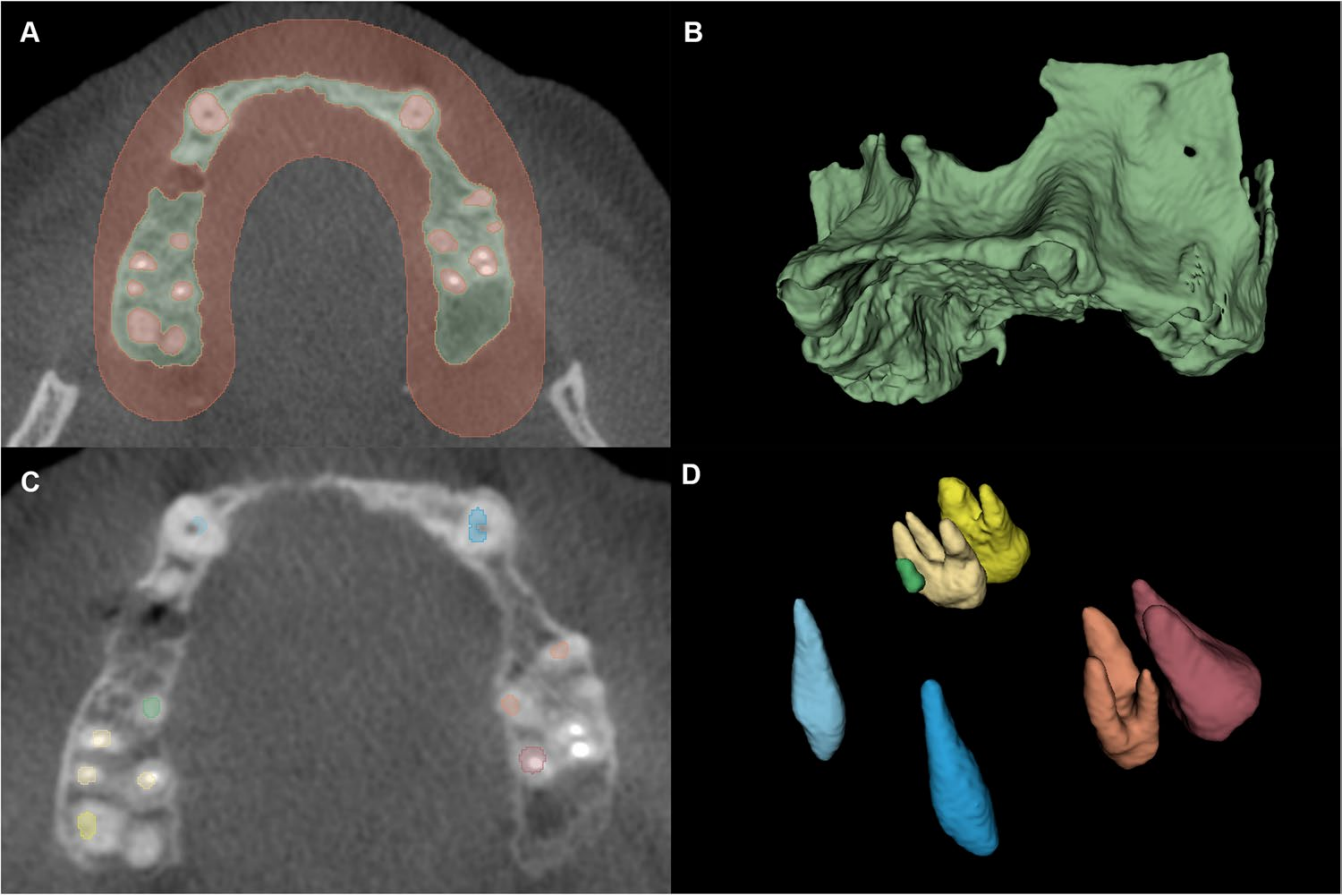
Presentation of the semi-automatic segmentation method. **A** Alveolar bone and background input labels generated on the axial view of the CBCT scan 3D model acquired via semi-automatic segmentation (green, alveolar bone; red, background). **B** 3D model of the alveolar bone following region-growing segmentation. **C** Labels generated for each tooth separately serves as the input of the watershed segmentation. **D** 3D surface representation of label maps following the application of the watershed segmentation method

An open-source medical image processing software platform (3D Slicer, www.slicer.org) [30] was utilized to reconstruct the T1, T2, and T3 CBCT images as 3D virtual models. A region-growing segmentation method was utilized to acquire 3D models of the maxillary alveolar bone. Two separate labels were generated: one for the maxillary alveolar bone and one for the background (including teeth). Seed points were placed on the planar images of the CBCT dataset both labels; thereafter, labels were generated automatically for the maxillary alveolar bone and the background activating the region-growing function (Fig. 4A and B). On T3 and T2 CBCT scans, the heads of the osteosynthesis screws were excluded from the label representing the bone volume; however, screw bodies were included. The reason is that the screw channel after removal of the screw can be considered as very small containing defect that has very limited impact on the outcomes.

Subsequently, teeth were segmented separately utilizing a watershed image segmentation method. Gray value intensity range of the teeth was determined, and seed points were drawn inside each individual tooth. Subsequently, the watershed segmentation tool was utilized to automatically generate a label for all teeth simultaneously (Fig. 4C and D) [31].

#### Evaluation of radiographic hard tissue changes

**Fig. 5:**
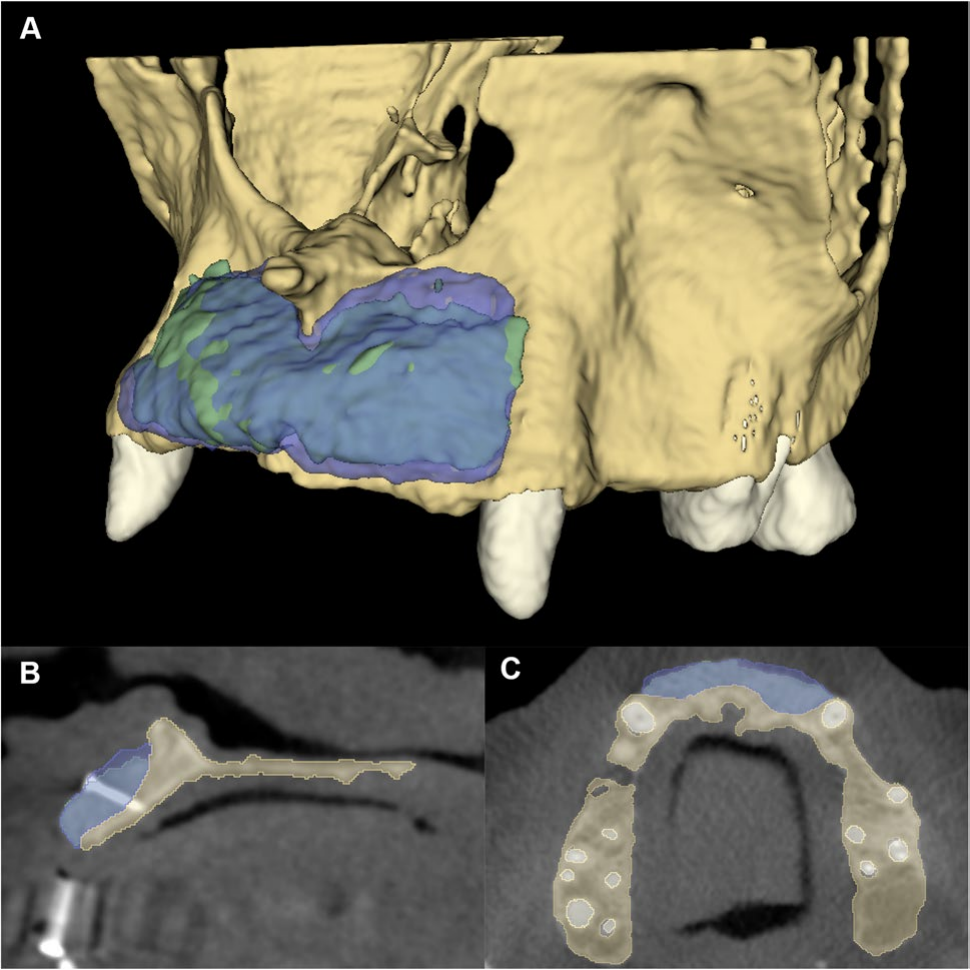
Volumetric changes between T1, T2, and T3 time points calculated with semi-automatic segmentation. **A** 3D visualization of the hard tissue gain (purple, 2-month control (T2); green, 6-month control (T3)). **B** Sagittal view. **C** Coronal view

Following image segmentation of all CBCT datasets, automatic voxel intensity-based registration was performed [32] to align the three CBCT scans in the virtual space. Thereafter, hard tissue changes between the three time points were determined by subtracting the segmented 3D models from one another by the means of logical operators. For example, to assess the volumetric gain between T2 and T1 time points, the T1 CBCT model was subtracted from the T2 CBCT model. Subsequently, 3D models of the new hard tissues at both T2 and T3 were generated (Fig. 5).

**Fig. 6:**
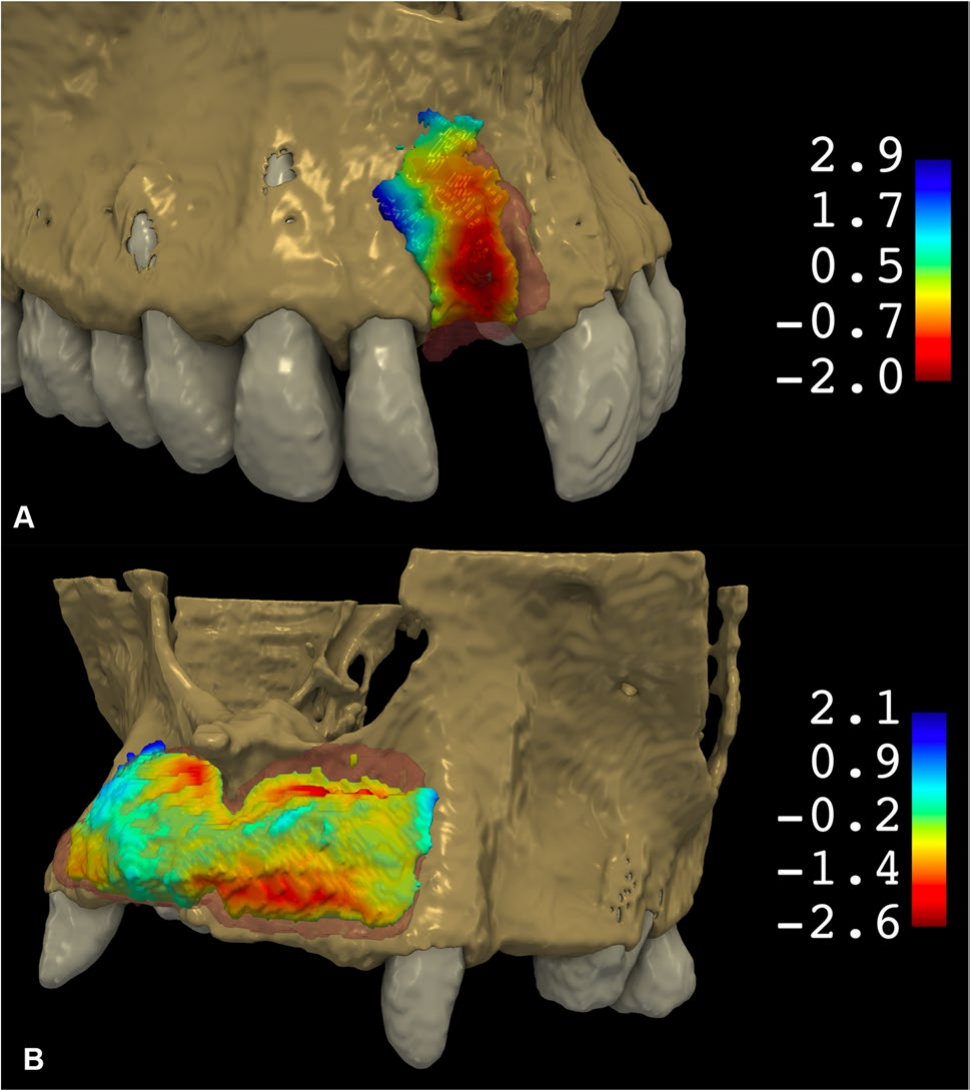
Three-dimensional morphological alterations between T2 and T3 visualized via a 3D colormap analysis (red, hard tissue loss; blue: hard tissue gain). **A** 3D hard tissue alterations between T2 and T3 at a single-tooth gap. **B** 3D hard tissue alterations between T2 and T3 at a multiple-tooth gap

The volume stability of the inserted allogeneic bone blocks was determined on the basis of the T3/T2 ratio (%) in both methods. Simultaneously, the dice similarity coefficient (DSC) was calculated to determine the spatial overlap between the models of newly formed hard tissue at T2 and T3 time points. The DSC was used to reflect how well the implanted CABB retained its original shape (Fig. 6).

Besides utilizing the aforementioned semi-automatic segmentation method, a global thresholding method was also utilized to evaluate outcomes. Results are described in the supplementary file.

Linear measurements at future implantation sites were performed to validate the vertical and horizontal dimensions of the edentulous crest at each corresponding time point. Linear measurement method and results are summarized in the supplementary file.

### Outcome measures

The primary outcome of the study was the volumetric hard tissue change between T1, T2, and T3 time points. The secondary outcomes were (i) volumetric stability of the CABB determined by the T3/T2 ratio, (ii) morphological stability expressed by the DSC value, and (iii) assessment of linear hard tissue dimensions at T1, T2, and T3 time points.

### Sample size calculation

Sample size was calculated by the means of power analysis using the G*Power statistical power analysis software (Heinrich Heine Universität Düsseldorf, Düsseldorf, Germany). Mean and standard deviation values of T2 and T3 volumetric gain values were determined as input parameters. The effect size (Cohen’s dz) was determined to be 0.55, and with *α* value of 0.05 and a power of 0.80, the total sample size was calculated to be 23.

### Statistical analysis

Descriptive statistics were used to describe the overall hard tissue changes among all participants. The overall changes were expressed as means ± standard deviations. The normality of the examined variables was checked with the Shapiro-Wilk test. Levene’s test checked the homogeneity of the variances. Data were found to be non-normally distributed, and variance homogeneity was also broken. The continuous variables between subgroups were compared with non-parametric statistics. The Wilcoxon matched pairs signed rank test was utilized to evaluate statistical differences for each variable at different time points. Correlation between the T3/T2 ratio and the DSC values was determined by Spearman rank order correlation test. The analysis was two sided with a significance level of *α* = 0.05. The statistical analysis was performed using the IBM® SPSS® 28.0 (IBM Corporation Armonk, NY, USA) program package.

## Results

### Patient demographics and baseline defect characteristics

Among the 23 participants, 14 were men, and 9 were women. The mean age was 45.48 ± 12.52 years. Single-tooth gaps were present in 13 participants, whereas 10 participants presented a multiple-tooth gap (two-tooth gap, 6 patients; three-tooth gap, 3 patients; and four-tooth gap, 2 patients). Linear measurements were taken at 40 future implantation sites.

According to the HVC ridge deficiency classification, 27 defects were classified as large horizontal defects, 6 as large combination defects, 6 as medium combination defects, and 1 as a medium horizontal defect. Data are summarized in Table 1.

**Table 1:** Patient demographic data and baseline defect characteristics

Participant	Sex	Age	Surgical area size	Tooth	HVC^3^
1	F^1^	34	2 teeth	11	CM^4^
				21	HL^5^
2	M^2^	38	1 tooth	21	HL
3	M	41	1 tooth	21	HL
4	F	53	1 tooth	21	HL
5	F	42	1 tooth	11	CM
6	F	47	1 tooth	11	CL^6^
7	M	44	2 teeth	22	HL
				21	HL
8	M	53	1 tooth	11	HL
9	F	42	4 teeth	12	HL
				11	HL
				21	CL
				22	HL
10	F	60	2 teeth	11	HL
				12	HL
11	M	55	2 teeth	12	HL
				11	HL
12	F	70	2 teeth	11	HL
				21	CM
13	F	48	1 tooth	12	HL
14	M	28	1 tooth	11	CM
15	M	72	3 teeth	12	HL
				11	HL
				21	HL
16	M	47	4 teeth	12	CL
				11	CL
				21	CL
				22	CM
17	M	55	1 tooth	21	CM
18	M	30	3 teeth	12	HL
				11	HL
				21	HL
19	M	20	1 tooth	12	HL
20	F	42	3 teeth	13	HL
				12	HL
				11	HM^7^
21	M	32	1 tooth	11	HL
22	M	48	1 tooth	21	CL
23	M	45	1 tooth	11	HL

^1^Female; ^2^male; ^3^horizontal, vertical, and combination ridge deficiency classification; ^4^combination medium defect; ^5^horizontal large defect; ^6^combination large defect; ^7^horizontal medium defect

### Volumetric hard tissue changes

At T2, an average of 0.75 cm^3^ ± 0.57 cm^3^ volumetric hard tissue gain was detected, with a median value of 0.49 cm^3^. At T3, an average of 0.52 cm^3^ ± 0.42 cm^3^ volumetric hard tissue gain could be detected with a median value of 0.37 cm^3^. As the result of the statistical analysis, a statistically significant volumetric hard tissue resorption was found between T2 and T3 (*p* < 0.05). Data are summarized in Table 2.

**Table 2:** Volumetric hard tissue changes (*n*=23)

	Mean ± St. Dev.^1^	Median	Min–max
New volume at T2^2^ (cm^3^)	0.75 ± 0.57	0.49	0.19–2.35
New volume at T3^3^ (cm^3^)	0.52 ± 0.42	0.37	0.09 ± 1.54
*p* value^4^	<0.05		

^1^Standard deviation, ^2^2-month follow-up, ^3^6-month follow-up, ^4^Wilcoxon matched pairs signed rank test

### Volumetric and morphological stability of CABBs

The average volume stability of the CABBs determined by the T3/T2 ratio was found to be 67.83% ± 18.72% on average with a median value of 72.46%. The DSC between the T2 and T3 hard tissue models averaged at 0.73 ± 0.15 with a median value of 0.77. High level of correlation between the T3/T2 ratio and the DSC value was found (Spearman’s correlation coefficient, 0.93, *p* < 0.05). These data are summarized in Table 3. As it is visible on the colormap models, hard tissue resorption is most expressed on the buccal/crestal aspect of the block (Fig. 5).

**Table 3:** Volumetric and morphological stability of CABB between T2 and T3 time points (*n*=23)

	Mean ± St. Dev.^1^	Median	Min–max
T3/T2 ratio (%)^2^	67.83 ± 18.72	72.46	19.57–95.83
DSC^3^	0.73 ± 0.15	0.77	0.32–0.89
Correlation coefficient^4^	0.93		
p value (T3/T2 – DSC)	*p* < 0.05		

^1^Standard deviation, ^2^describing the volume stability of the graft, ^3^dice similarity coefficient, ^4^Spearman rank order correlation test

## Discussion

In the current study, 23 advanced alveolar ridge deficiencies in the upper anterior region were reconstructed using CABBs. Volumetric radiographic evaluation was performed by comparing segmented 3D CBCT datasets in different time points. The primary outcome of the study was to evaluate the volumetric gain achieved over the course of 6-month healing period. Additionally, the current paper focused on assessing the volumetric and morphological stability of the implanted CABB between T2 and T3 time points. At T2, the average new hard tissue formation was found to be 0.75 cm^3^ ± 0.57 cm^3^ which reduced to 0.52 cm^3^ ± 0.42 cm^3^ at T3 resulting in an approximately 32% volumetric hard tissue This resorption rate is similar to previously reported data on cancellous allogeneic bone blocks—approximately 29% [33]. To our knowledge, no data was reported on the spatial overlap of the inserted bone block between two different time points. This metric, expressed by the DSC value, was found to be 0.73 ± 0.15 on average. The DSC values showed high levels of correlation to the T3/T2 ratio indicating the volumetric stability. Resorption patterns are difficult to express numerically, even though from a clinical point of view this observation is very important, since the interpretation of these results may lead to the enhancement of the surgical techniques and the development of new surgical materials. Similarly to the volumetric data, a significant linear vertical and horizontal hard tissue resorption was detected between T2 and T3. In the study by Wang and co-workers, horizontal resorption of the corticocancellous allogeneic graft averaged at 2.28 mm ± 1.14 mm, and vertical hard tissue dimension loss averaged at 1.77 mm ± 0.95 mm. Whereas in the current investigation, the horizontal alveolar ridge dimensions were reduced by about 1.4–1.5 mm, the vertical alveolar ridge dimensions at the treated sites were reduced by about 0.5–0.7 mm. The implanted cancellous CABBs herein presented similar or less dimensional loss than did those in the studies by Tresguerres et al. and Wang et al. [19, 33]. Within the limitations of the current study, the reasons for the more favorable outcomes would be difficult to determine. However, it can be emphasized that the limited flap elevation and the semi-pillar incision facilitated tension-free covering of the grafts. The high-precision CAD/CAM of the CABBs may have also contributed to the seemingly better results in this study [22]. The current volumetric and graft stability data are in line with the literature.

The volumetric changes were evaluated using two methods, although these methods showed a high correlation with. Difference between the two methods could be attributed to the fact that the algorithm of global thresholding segmentation automatically labels voxels that fall within the threshold range. Contrary to the semi-automatic segmentation, this method does not recognize anatomical features and artifacts on CBCT scans. Meanwhile, during semi-automatic segmentation, the input data for region-growing and watershed algorithms are generated manually by a human. Nonetheless, both methods were found to be feasible for the volumetric evaluation of hard tissue changes, although utilizing 3D Slicer served as a much more elaborative approach.

The greatest limitation of the current study is the relatively large diversity of the baseline defect morphologies. Although all cases presented a horizontal alveolar ridge deficiency, the baseline horizontal ridge dimensions varied greatly (1.49–6.43 mm). Simultaneously, not all future implantation sites presented a vertical dimension loss, with only 12 being categorized as combination defects according to the HVC classification.

## Conclusions

The current findings support previous literature data. Therefore, it can be concluded that cancellous CAD/CAM allogeneic bone blocks can be reliably utilized for the reconstruction of severely atrophied alveolar ridges. The resorption rates of these grafts are similar to those found in the literature; however, with precise manufacturing and proper intraoperative flap management, the rates may be reduced. Although morphological alterations are difficult to express numerically, these findings could contribute to the future development of surgical techniques and regenerative materials.

## Supplementary Information

ESM 1: Supplementary File. Efficacy and volume stability of a customized allogeneic bone block for the reconstruction of advanced alveolar ridge deficiencies at the anterior maxillary region: A retrospective radiographic evaluation
