# Supplementary material for: Efficacy and volume stability of a customized allogeneic bone block for the reconstruction of advanced alveolar ridge deficiencies at the anterior maxillary region: a retrospective radiographic evaluation
Source: Clin Oral Investig. 2023 Apr 14;27(7):3927–35. doi: 10.1007/s00784-023-05015-0 (PMC10329613; doi:10.1007/s00784-023-05015-0)
Supplement: Supplementary file 1 — Supplementary File. Efficacy and volume stability of a customized allogeneic bone block for the reconstruction of advanced alveolar ridge deficiencies at the anterior maxillary region: A retrospective radiographic evaluation [file 784_2023_5015_MOESM1_ESM.docx]

**Supplementary File**

**Efficacy and volume stability of a customized allogeneic bone block for the reconstruction of advanced alveolar ridge deficiencies at the anterior maxillary region: A retrospective radiographic evaluation**

**Introduction**

In the present study volumetric evaluation following alveolar ridge augmentation with a customized allogeneic bone block (CABB) was performed by the means of 3D subtraction analysis. Cone-beam computed tomography (CBCT) scans take at three different timepoints (baseline: T1, 2-month follow-up: T2, 6-month follow-up: T3) were reconstructed in 3D utilizing radiographic image segmentation. Besides semi-automatic segmentation - as described in the main body of the manuscript – a global thresholding segmentation method was also utilized to determine volumetric hard tissue changes between T1, T2 and T3 timepoints. Additionally to volumetric measurements, vertical- and horizontal dimensions at future implantation sites were evaluated by linear measurements at T1, T2 and T3 timepoints.

The aim of presenting additional evaluation methods, was to provide even further details on hard tissue alterations that occurred following surgery.

**Materials and methods**

*Global thresholding segmentation method*

Three-dimensional models of T1, T2, and T3 CBCT scans were acquired using a global thresholding segmentation method in the coDiagnostiX software. Landmark-based image registration was performed to spatially align the three CBCT datasets. Voxel gray-levels were selected to mark the upper- and lower boundaries of the reconstruction. Subsequently, one labelmap was generated on the voxels that fall between the previously set boundaries. A 3D surface representation of the 2D labelmap was automatically generated. Volumetric differences between T1, T2, T3 timepoints were calculated mathematically (Supplementary Figure 1.).

*Linear hard-tissue changes*

On the T3 CBCT scans, prosthetically driven implant positions were marked using radiopaque radiographic markers. At the future implantation sites, absolute horizontal and vertical linear dimensions of the edentulous ridge were registered on the T1, T2, and T3 CBCT scans in 3D Slicer. The CBCT datasets were reoriented such that the coronal plane became parallel, and the axial plane became perpendicular to the long axis of the edentulous ridge. A vertical linear dimension of the alveolar ridge was measured from the midcrestal point along the long axis of the alveolar ridge (marked by the coronal plane) to the base of the nasal cavity. Meanwhile, horizontal linear measurements were performed 2 mm apical to the alveolar crest at the same aspect perpendicular to the long axis of the alveolar ridge (marked by the axial plane) between the palatal and buccal cortical plates (Supplementary Figure 2.).

**Results**

*Volumetric hard tissue changes – Global thresholding method*

At T2, an average of 0.69 cm^3^ ± 0.56 cm^3^ volumetric hard-tissue gain was detected, with a median value of 0.46 cm^3^. At T3, an average of 0.53 cm^3^ ± 0.46 cm^3^ volumetric hard tissue gain could be detected with a median value of 0.37 cm^3^. As the result of the statistical analysis a statistically significant volumetric hard tissue resorption was found between T2 and T3 (*p* < 0.05). The average volume stability of the CABBs determined by the T3/T2 ratio was found to be 75.50% ± 13.68% on average with a median value of 76.47%. Data are summarized in Supplementary Table 1.

*Correlation between the two volumetric evaluation methods*

Compared to results measured with the semi-automatic segmentation method at T2, a statistically significant difference could be detected with the Wilcoxon matched pairs signed rank test (*p* = 0.009), although high level of correlation could be detected between the two metrics (Spearman correlation coefficient: 0.95). Contrary, statistically significant difference between the semi-automatic- and the global thresholding segmentation methods could not be detected regarding the volumetric hard tissue gain at T3 (*p* = 0.89). High levels of correlation was also found between the two datasets regarding this metric (Spearman correlation coefficient: 0.91). Data are summarized in Supplementary Table 2.

*Linear hard tissue dimensions*

The baseline vertical alveolar ridge dimensions averaged at 15.45 mm ± 3.32 mm at future implantation sites, whereas baseline horizontal ridge dimensions averaged at 3.30 mm ± 1.04 mm. At T2 the average linear vertical dimension at future implantation sites averaged at 17.60 mm ± 2.82, horizontal ridge dimensions were measured at an average of 7.85 mm ± 1.14 mm. At T3 vertical- and horizontal ridge dimensions averaged at 16.97 mm ± 2.86 mm and 6.43 mm ± 1.27 mm respectively. Statistically significant differences were recorded between all the metrics. Resulting in a statistically significant vertical- and horizontal linear gain between T1 and both follow-up timepoints (T2 and T3). Simultaneously a statistically significant linear hard tissue loss could be detected between T2 and T3. These data are summarized in Supplementary Table 3.

**Discussion**

The volumetric changes were evaluated using two methods, although these methods showed a high correlation, however with varying significance level detected between T2 and T3 data. Volumetric results at T2 timepoint calculated with the semi-automatic and global thresholding methods were statistically significant (*p* = 0.009), whereas at T3 timepoint no statistically significant difference could be detected between the results of the two datasets (*p* = 0.89). This finding could be attributed to the fact that the algorithm of global thresholding segmentation automatically labels voxels that fall within the threshold range. Global thresholding segmentation algorithms do not recognize anatomical features and cannot differentiate artifacts on CBCT scans. Meanwhile, during semi-automatic segmentation, the input data for region-growing and watershed segmentation algorithms are generated manually by a human. The disadvantage of semi-automatic segmentation is the relative time-consuming process of generating the input labelmaps of all the anatomical structures separately.

Nonetheless, both methods were found to be feasible for the volumetric evaluation of hard-tissue changes, although utilizing 3D Slicer served as a much more elaborative approach.

**Tables**

| **Supplementary Table 1: Volumetric hard tissue changes recorded with global thresholding segmentation (n=23)** | | | |
| --- | --- | --- | --- |
|  | Mean ± St. Dev.^1^ | Median | Min - Max |
| New volume at T2^2^ (cm^3^) | 0.69 ± 0.56 | 0.46 | 0.19 - 2.24 |
| New volume at T3^3^ (cm^3^) | 0.53 ± 0.46 | 0.37 | 0.11 - 2.05 |
| T3/T2 ratio (%) | 75.50 ± 13.68 | 76.47% | 46.15 - 97.87 |
| *p* value^4^ | < 0.05 | | |
| ^1^standard deviation, ^2^2-month follow-up, ^3^6-month follow-up, ^4^Wilcoxon matched pairs signed rank test between (significance level: p<0.05) “New volume at T2” and “New volume at T3” | | | |

| **Supplementary Table 2: Comparison of results acquired with the semi-automatic segmentation and the global thresholding segmentation methods (n=23)** | | | | | | |
| --- | --- | --- | --- | --- | --- | --- |
|  | Semi-automatic segmentation | | Global thresholding segmentation | | *p* value^2^ | Spearman Correlation coefficient |
|  | Mean ± st. dev.^1^ | Median | Mean ± st. dev. | Median |  |  |
| New volume at T2^3^ (cm^3^) | 0.75 ± 0.57 | 0.49 | 0.69 ± 0.56 | 0.46 | =0.009 | 0.95 |
| New volume at T3^4^ (cm^3^) | 0.52 ± 0.42 | 0.37 | 0.53 ± 0.46 | 0.37 | =0.89 | 0.91 |
| ^1^standard deviation ,^2^Wilcoxon matched pairs signed rank test (significance level: p<0.05), ^3^2-month follow-up, ^4^6-month follow-up | | | | | | |

| **Supplementary Table 3: Vertical linear dimension at future implant positions (n=40)** | | | |
| --- | --- | --- | --- |
|  | Mean ± St. Dev.^1^ | Median | Min - Max |
| Vertical dimension T1^2^ (mm) | 15.45 ± 3.32 | 15.15 | 9.06 - 21.95 |
| Vertical dimension T2^3^ (mm) | 17.60 ± 2.82 | 18.22 | 11.34 -21.53 |
| Vertical dimension T3^4^ (mm) | 16.97 ± 2.86 | 17.53 | 12.02 - 21.42 |
| *p* value^5^ (T1 - T2) | < 0.05 | | |
| *p* value5 (T2 - T3) | < 0.05 | | |
| *p* value5 (T1 - T3) | < 0.05 | | |
| ^1^standard deviation, ^2^baseline, ^3^2-month follow-up, ^4^6-month follow-up, ^5^Wilcoxon matched pairs signed rank test (significance level: p<0.05) | | | |

| **Supplementary Table 4: Horizontal linear dimension at future positions (n=40)** | | | |
| --- | --- | --- | --- |
|  | Mean ± St. Dev.^1^ | Median | Min - Max |
| Horizontal dimension T1^2^ (mm) | 3.30 ± 1.04 | 3.32 | 1.49 - 6.43 |
| Horizontal dimension T2^3^ (mm) | 7.85 ± 1.14 | 7.61 | 5.93 - 10.32 |
| Horizontal dimension T3^4^ (mm) | 6.43 ± 1.27 | 6.53 | 3.61 - 9.33 |
| *p* value^5^ (T1 - T2) | < 0.05 | | |
| *p* value5 (T2 - T3) | < 0.05 | | |
| *p* value5 (T1 - T3) | < 0.05 | | |
| ^1^standard deviation, ^2^baseline, ^3^2-month follow-up, ^4^6-month follow-up, ^5^Wilcoxon matched pairs signed rank test (significance level: p<0.05) | | | |

**Figure legends:**

**Supplementary Figure 1:** Presentation of the global thresholding segmentation method

**A:** 3D model acquired via global thresholding segmentation (blue: baseline alveolar ridge, purple: hard-tissue gain at the 6-month follow-up)

**B:** Axial view of the labelmaps generated using global thresholding segmentation (orange: baseline alveolar ridge morphology, pink: hard-tissue gain at the 6-month follow-up, red outline: hard-tissue gain at the 2-month follow-up)

**Supplementary Figure 2:** Vertical and horizontal hard-tissue measurements at the implantation sites

**A:** Baseline (T1)

**B:** 2-month follow-up (T2)

**C:** 6-month follow-up (T3)
